# Fatal Uterine Hemorrhage, the Result of Professional Inactivity

**Published:** 1866-09

**Authors:** J. M. Owens

**Affiliations:** Hamburg, Ark.


*Fatal Uterine Hemorrhage, the result of professional inactivity.*

By J. M. Owens, M. D., Hamburg, Ark.

I give a brief report of uterine hemorrhage terminating fatally for want of timely assistance, or from painful inactivity on the part of those in attendance.

Mrs. ———, aged about twenty-six years, of sanguine temperament and of unexceptionable constitution, was confined twice previous to the confinement which took place in December last, and which proved fatal.

On the 28th of February, 1861, she was delivered of her first child, at which time she was brought almost to death's door, by an alarming hemorrhage. The labor was tedious and difficult, on account of a face presentation; and in addition there proved to be adherent placenta, that appendage being attached high up in the fundus, the funis being coiled around the neck of the child.

The child was born asphyxiated, but was resuscitated after an hour's rigid application of Hall's method, the major part of which had to be entrusted to the care of the nurse (who happened to be quite an intelligent lady), my own attention being demanded by the mother. I immediately proceeded to remove the placenta by introducing the hand and gently breaking up the adhesions, the uterus being in a state of inertia and bleeding profusely. By persevering in the use of cold application and decoction of ergot, together with mustard to the spine, and pressure applied with compress and bandage over the region of the womb, in six hours the hemorrhage was entirely arrested, and I had the satisfaction of seeing the mother make a good recovery. The child also did well. On the 4th of December, 1863, Mrs. ——— was again confined. I was called, and arrived at her bed-side before the contractions become very severe. She was greatly alarmed, however, saying she could not possibly survive her confinement if she must suffer as in her first labor. Her fears were relieved when, after an examination, I informed her this was a natural presentation and that she would have a comparatively easy time.

My opinion was verified by the result; the labor lasting only four hours. But, on immediately introducing the hand, (which I was warned to do by the adherent placenta and alarming hemorrhage in her previous confinement) I found just such a state of things existing as before, with an hour-glass contraction already beginning; the placenta was easily detached and delivered, giving the womb an opportunity to contract, thereby preventing the possibility of hemorrhage.

On the 10th of December, Mrs. ——— was confined the third time. Her husband called on me in October, to request me to hold myself in readiness to attend her. At this time he lived fourteen miles distant. I urged on him the propriety of sending Mrs. ——— to town; but it was not convenient for her to leave home. I warned him to be careful, and, in case of sickness preventing my compliance with her request, to be certain to have a physician of some obstetrical experience and good judgment, with a mind prompt to act; and I requested him to tell the physician in attendance, for me, to remove the placenta as soon as the child was taken charge of by the nurse, and to be certain not to wait to remove the placenta by traction on the cord, if it offered the least resistance, but to introduce the hand far up, as on its speedy removal depended her existence.

On the 9th of December a boy came in great haste for me to see Mrs. ———. I was confined to my bed by sickness. I tried to get my confreres to attend the case, but they were either engaged or unable. I dressed and started, but found, on driving three miles, that I could not proceed. There were physicians in the neighborhood, and I supposed she would be well cared for. Early on the morning of the 10th, a second messenger came to say Mrs. ——— was very ill. I started again, and when in three miles of the residence I met a servant coming with the measure of Mrs. ———'s coffin.

I saw her husband, and he told me with tearful eyes, that the labor was not a difficult or protracted one, but, that the placenta was not removed. A physician living near was called in, and arrived soon after the child was born, Mrs. ——— requested the Doctor to remove the placenta, stating that I had removed it with but little trouble on two occasions immediately after delivery, and that unless it was speedily removed she would bleed to death. The physician failed to do as requested (from what cause I am not prepared to say) and as might have been expected under the circumstances, in a few hours Mrs. ——— was dead. It may be well to state, in this connection, that the first wife of Mr. ——— (the subject of this report being the second) had several violent hemorrhages from adherent placenta, and died of inflammation produced from that cause, the placenta not being yet delivered when she died nine days after miscarriage at four months of gestation.

I have been in the habit for several years (and I think I am justified in the practice) of removing the placenta immediately after delivery, and if I fail by gentle traction on the cord, and by unbuttoning as you would unbutton your coat, I immediately proceed to introduce the hand, while the parts are yet dilated, and deliver the placenta at once. Most authors tell us to wait an hour after the child is born, or until the uterus begins to contract. In the great majority of cases, doubtless, the placenta will be detached and expelled by the efforts of the uterus, nothing more being required than gentle traction; but if there should be adhesion to the uterine walls, and you wait an hour, or until the uterus begins to contract, the vagina in the meantime contracting, gradually assuming its natural dimensions and becoming tender, you are losing valuable time, and independent of hour-glass contraction, which frequently happens in adherent placenta, you will find very great difficulty in introducing the hand without the use of chloroform, which is certainly not devoid of danger in a subject already prostrated; whereas, immediately after delivery, when the organs are thoroughly dilated and the mother in high spirits and full of hope, rejoicing at the favorable issue of her confinement, it is easy, in a large proportion of cases, to introduce the hand and detach the adherent placenta with little pain or inconvenience to the mother, and no trouble to the physician, in doing which you have accomplished two very desirable objects, viz., saved the patient much unnecessary suffering, and prevented serious hemorrhage, or a painful, protracted, and possibly fatal, metritis.—*Southern Journal of the Medical Sciences.*

